# Supplementary material for: TFAP2C is a key regulator of intrauterine trophoblast cell invasion and deep hemochorial placentation
Source: JCI Insight. 2024 Dec 3;10(2):e186471. doi: 10.1172/jci.insight.186471 (PMC11790029; doi:10.1172/jci.insight.186471)
Supplement: Supplemental data [file jciinsight-10-186471-s089.pdf]

Supplemental material

TFAP2C is a key regulator of intrauterine trophoblast cell invasion and deep hemochorial placentation

Esteban M. Dominguez[1,*], Ayelen Moreno-Irusta[1], Regan L. Scott[1], Khursheed Iqbal[1,†], and Michael J. Soares[1,2,3,*]

[1]Institute for Reproductive and Developmental Sciences, Department of Pathology & Laboratory Medicine, University of Kansas Medical Center, Kansas City, Kansas 66160, [2]Department of Obstetrics and Gynecology, University of Kansas Medical Center, Kansas City, Kansas 66160, [3]Center for Perinatal Research, Children's Mercy Research Institute, Children's Mercy, Kansas City, MO 64108

[†]Present address: Department of Animal and Food Sciences, Oklahoma State University, Stillwater, Oklahoma

***Correspondence:** edominguez@kumc.edu **or** msoares@kumc.edu

Supporting Figures and Tables

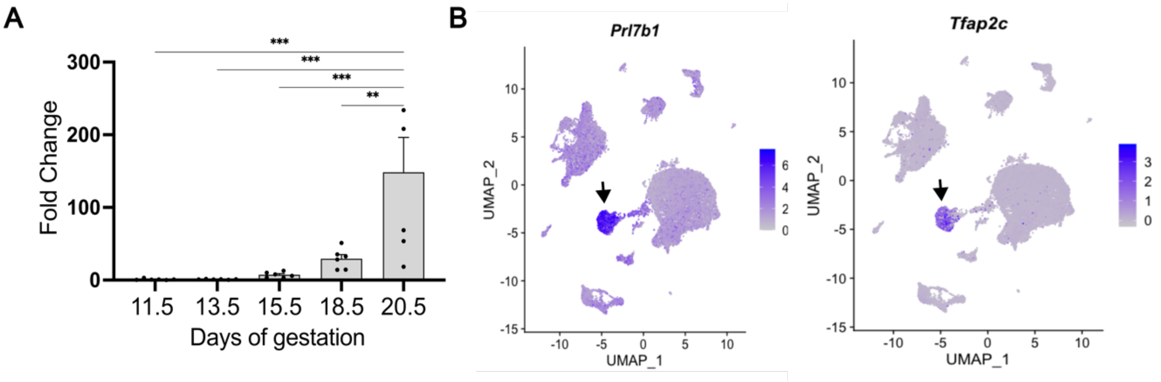

Supplemental Figure 1. *Tfap2c* transcript expression in the rat uterine-placental interface. A) RT-qPCR of *Tfap2c* transcript in uterine-placental interface tissues on gestation days 11.5, 13.5, 15.5, 18.5, and 20.5. Data are expressed as mean ± standard error of the mean. Each data point represents a biological replicate from six different pregnancies (n=6). Unpaired *t*-test: **p<0.01, ***p<0.001. **B**) Uniform manifold approximation and projection plot (UMAP) for *Prl7b1* and *Tfap2c* transcripts from single cell RNA sequencing of the rat uterine-placental interface at gestation day 19.5 (38). Note the co-localization of *Prl7b1* and *Tfap2c* transcripts in the invasive trophoblast cell cluster (arrows).

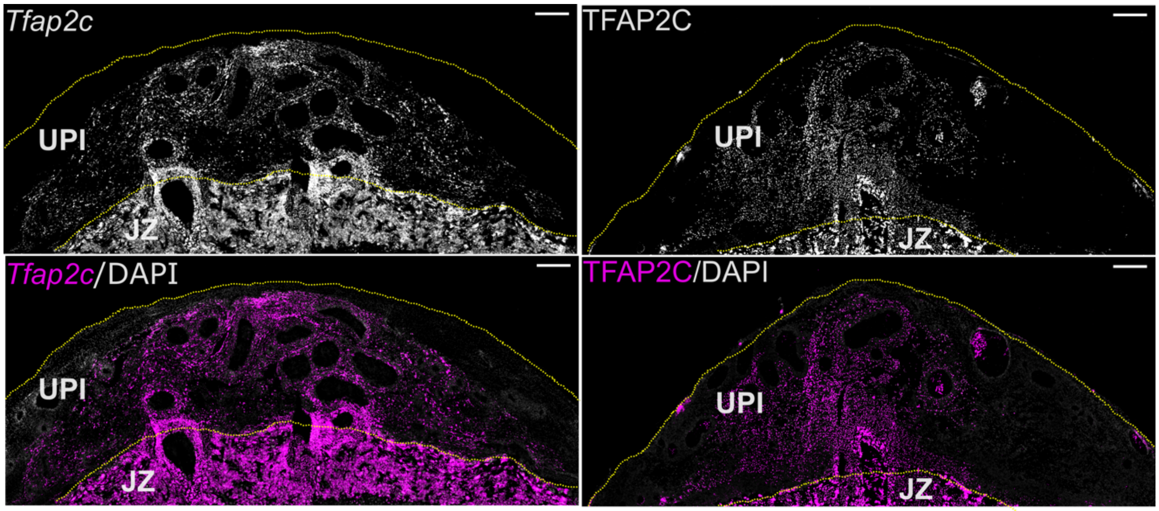

Supplemental Figure 2. TFAP2C transcript (left panel) and protein (right panel) localization within the gestation day 18.5 uterine-placental interface. TFAP2C positive cells (magenta) are distributed within the junctional zone (JZ) and uterine-placental interface (UPI). Scale bar: 500 μm.

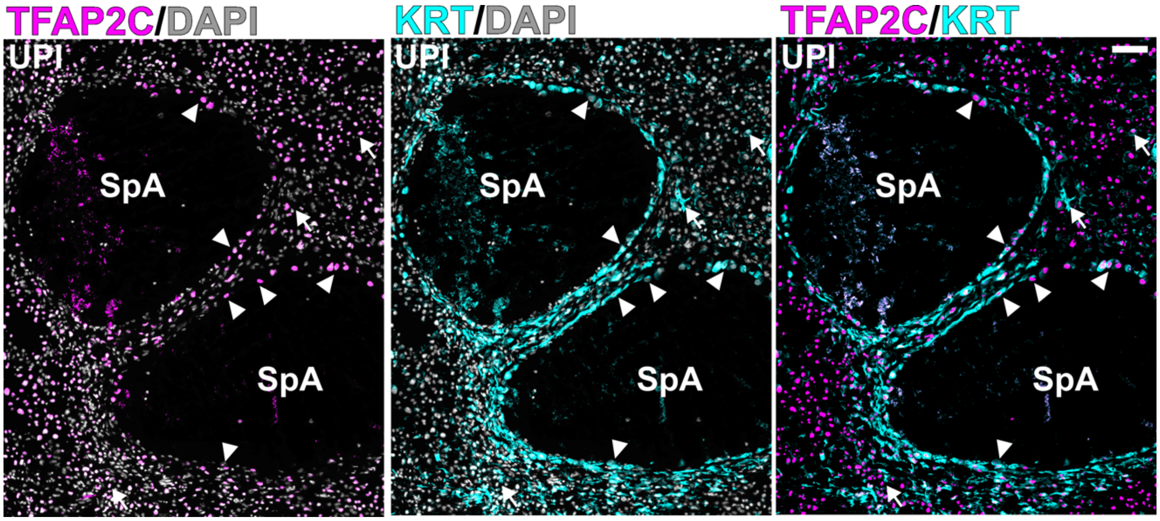

Supplemental Figure 3. TFAP2C protein localization in the uterine-placental interface. Immunohistochemistry of TFAP2C (magenta) and cytokeratin (KRT, cyan) within the rat uterine-placental interface at gd 18.5. TFAP2C was localized in nuclei of both endovascular (arrowhead) and interstitial (arrow) invasive trophoblast cells. Abbreviations, UPI, uterine-placental interface; SpA, spiral artery. Scale bar: 100 μm

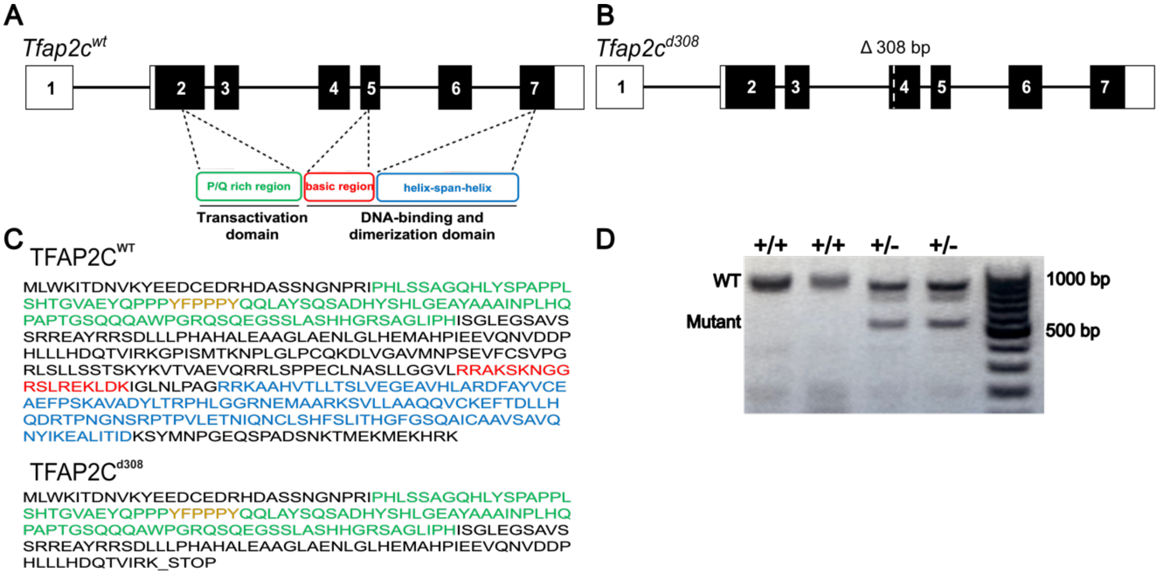

Supplemental Figure 4. Global disruption of *Tfap2c*. A) Schematic of *Tfap2c* exon-intron structure and encoded functional domains within the TFAP2C protein. **B**) Schematic of the *Tfap2c* gene possessing a 308 bp deletion (*Tfap2c*^{d308}). **C**) Amino acid sequences corresponding to wild type TFAP2C (TFAP2C^{WT}) and the amino acid sequence for mutant TFAP2C (TFAP2C^{d308}). **D**) Genotyping of *Tfap2c*^{WT} and heterozygous *Tfap2c*^{d308} conceptuses.

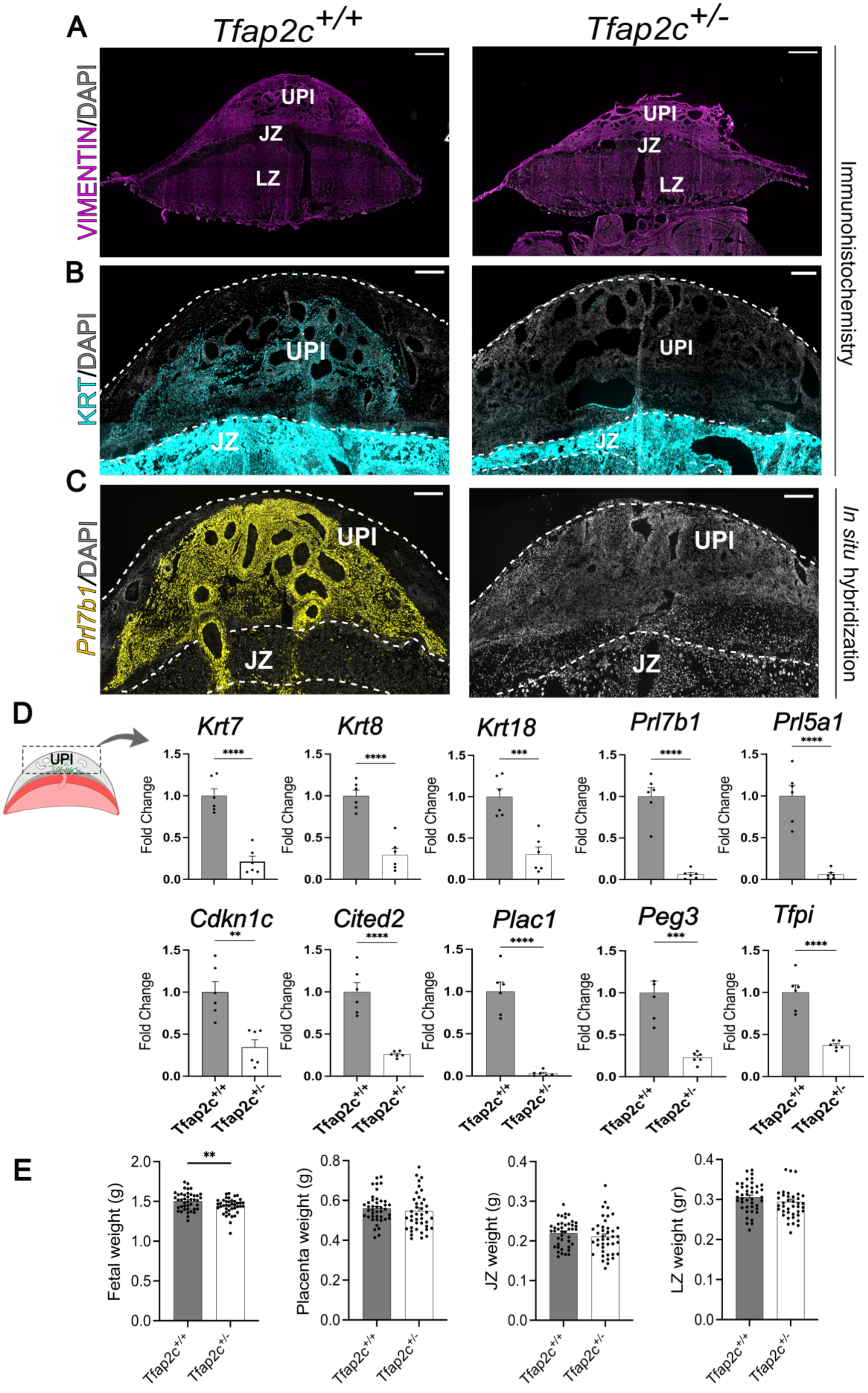

Supplemental Figure 5. *Tfap2* gene dosage effects on placenta development. Placentation sites were generated from female *Tfap2c*$^{+/-}$ x male *Tfap2c*$^{+/+}$ breeding. **A**)

Identification of placentation site compartments using vimentin immunostaining on gestation day (**gd**) 18.5 *Tfap2c*$^{+/+}$ and *Tfap2c*$^{+/-}$ placentation sites (magenta). **B**) Immunohistochemical localization of cytokeratin protein in gd 18.5 *Tfap2c*$^{+/+}$ and *Tfap2c*$^{+/-}$ placentation sites (cyan). **C**) Distribution of *Prl7b1* transcripts in gd 18.5 *Tfap2c*$^{+/+}$ and *Tfap2c*$^{+/-}$ placentation sites (yellow). **D)** RT-qPCR of invasive trophoblast cell-specific transcripts in gd 18.5 *Tfap2c*$^{+/+}$ and *Tfap2c*$^{+/-}$ uterine-placental interface tissues. Data are expressed as mean ± standard error of the mean (SEM). Each data point represents a biological replicate from six different pregnancies (n=6). **E**) Fetal, placenta, junctional zone and labyrinth zone weights from gd 18.5 *Tfap2c*$^{+/+}$ and *Tfap2c*$^{+/-}$ conceptuses. Data are expressed as mean ± SEM. Each data point represents a biological replicate from six different pregnancies (*Tfap2*+$^{+/+}$, n=41; *Tfap2c* $^{+/-}$, n=42). Unpaired *t*-test: **p<0.01, ***p<0.001, ****p<0.0001. Abbreviations: UPI, uterine-placental interface; JZ, junctional zone; LZ, labyrinth zone; SpA, spiral artery. Scale bars: 500 μm.

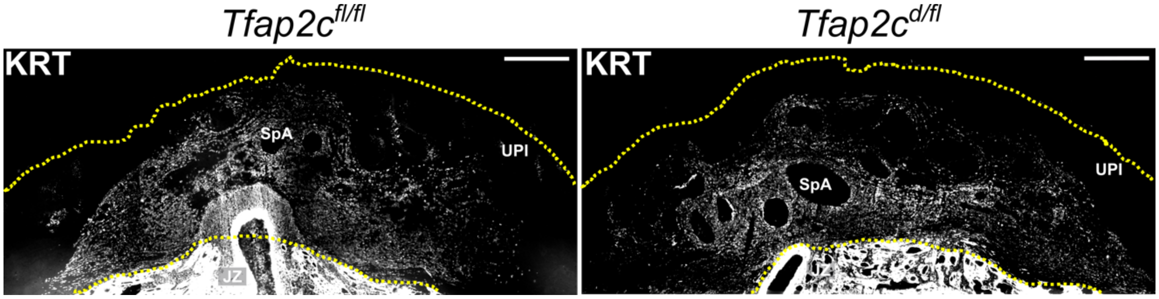

Supplemental Figure 6. Invasive trophoblast cell distribution within the uterine-placental interface of the *Tfap2c$^{fl/fl}$* and *Tfap2c$^{d/fl}$* placentation sites. Invasive trophoblast cells were localized in the uterine-placental interface using immunocytochemistry for cytokeratin (white). Abbreviations, SpA, spiral artery; UPI, uterine-placental interface; JZ, junctional zone. Scale bar: 500 μm.

Supplemental Table 1. Genotyping results from female *Tfap2c*$^{+/-}$ x male *Tfap2c*$^{+/-}$ matings.

Samples	*Tfap2c* genotype		
Gestation day:	+/+ (%)	+/- (%)	-/- (%)
8.5	3 (14)	13 (59)	6 (27)
9.5	7 (22)	25 (78)	0 (0)
12.5	6 (32)	13 (68)	0 (0)
15.5	23 (41)	33 (59)	0 (0)
18.5	20 (34)	38 (66)	0 (0)
Postnatal day 1	23 (48)	25 (52)*	0 (0)

*3 *Tfap2c*$^{+/-}$ pups found dead.

Supplemental Table 2. Genotyping results from female *Tfap2c*$^{+/-}$ x male *Tfap2c*$^{+/+}$ matings.

Samples	*Tfap2c* genotype	
Gestation day:	+/+ (%)	+/- (%)
15.5	38 (46)	44 (54)
18.5	41 (51)	40 (49)
Postnatal day 1	24 (53)	21 (47)

Supplemental Table 3. Genotyping results from female *Tfap2c*$^{+/+}$ x male *Tfap2c*$^{+/-}$ matings.

Samples	*Tfap2c* genotype	
Gestation day:	+/+ (%)	+/- (%)
15.5	33 (60)	22 (40)
18.5	34 (62)	28 (45)
Postnatal day 1	22 (61)	14 (39)

Supplemental Table 4. Guide RNAs used for genome editing.

gRNA	DNA sequence
Tfap2c (Exon4)	GGTTCATGACCGCTCCAACTGTTTTAGAGCTATGCT
Tfap2c (5' of Exon 4)	TTAAGTTATATACACTGACAGTTTTAGAGCTATGCT
Tfap2c (3' of Exon 4)	TTAAGGCCGCAGCTGCATGGGTTTTAGAGCTATGCT

Supplemental Table 5. Template sequences used for genome editing.

Tfap2c 5' loxP template (5' of Exon 4)
CTTAATGAGGGGTTTGTAGGAGGGAGGAGATGGGGGGAATCGGATTACAGT<br>GAGTGGGTGGCTCTTGCTTAAGTTATATACACTGATAACTTCGTATAGCATAC<br>ATTATACGAAGTTATACACGGTTACCGGATATGGAATCGCCCTAAATTACAG<br>GTAGTTATGGTCTTGAGATTGTGAACTGTATTAGTAAATTGGGA
Tfap2c 3' loxP template (3' of Exon 4)
TTCTTTTTGAAAGTGCCAAAATGCAGAAGGTGCTGAAACAGCATAACAGGTT<br>ATCATTTGGTTGGGATTAAGGCCGCAGCTGCAATAACTTCGTATAGCATACA<br>TTATACGAAGTTATTGGAGGGGAGGGGTGGCTTCGAGTGTTTGGTCCCCCT<br>GGGGGAGTTTACTGCTACAATCCTAAGTGAAGATTTTTCTGCACC

Supplemental Table 6. Primer sequences used for genotyping.

Primer Name	Forward Primer	Reverse Primer
Tfap2c	AGGGGGAGGATGCCATTTAT	GGGGACCAAACACTCGAAG
5' Floxed Tfap2c	GGGTTTGTAGGAGGGAGGAG	GGTCTGCTCAGAAACAGTTTTC
3'Floxed Tfap2c	AAGGTGCTGAAACAGCATAACA	GGCCTCCATTTTTGGATTTC
Prl7b1Cre	TGCTGGAAGATGGCGATTAG	GGTTCCCAGCAAAGTACCAC
Kdm5d	TTGGTGAGATGGCTGATTCC	GGTTTCTTAAACCGTCGCC
Kdm5c	TTTGTACGACTAGGCCCCAC	GGTTTCTTAAACCGTCGCC

Supplemental Table 7. Primer sequences used for RT-qPCR.

Primer Name	Forward Primer	Reverse Primer
Peg3	AAGTTCACGTCCACTCCGTC	CGTCTGGTCTTGTTCGTGGA
Plac1	CCGTCTCTCCAGATGTCGTT	GAGCCCTTGGAAGCATAGTG
Cited2	TCTTGGCTGCATGAACTTTG	GGGAGACAGCCAACTTGAAA
Cdkn1c	CAAACGTCTGCGATGAGTTAGT	AGCCGAAGCCCAGAGTTC
Tfpi	GCCCGAGGAAGACGATGATA	TCCGCCTTCATTGCACAG
Prl5a1	TCCACACCAGACATTCCAGA	TTTCCAGGAAGCCAACATTC
Prl7b1	CCGTCATACTGTCTCAGCACATC	AGCTGTTGAGACCATTGACAACAA
Gapdh	GACATGCCGCCTGGAGAAAC	AGCCCAGGATGCCCTTTAGT
Krt7	CGGAATGGGACCTGTGAA	GTAGATGTGGTCTTGATGGAATAGG
Krt8	TGGGCCAGGAGAAGCTGAA	CACATCCTTCTTGATGAGGACAAA
Krt18	CTGGAAACCGAGAACAGGAGA	CGGGCATTGTCCACAGAA
Prf1	GGCACTCAAGAACCTTCC	CTCAAGCAGTCTCCTACC
Klrb1c	GTCTCCAGGGCATAAGCAAG	AAGAAGGATCAGCGTAGCACA
Klrb1a	CGTTCACACAGGTTGGCTTT	TGGCTCCACTGATGGTTTTT
Klrb1	GGTCTCGCTGACTGTTCTTTG	TTGTTGTCCTTTTCCCTTTG
Ncr1	ATGGGAACATCCAAGCAGAG	ACAGGCTCACTGGGAAAAGA
