# Supplementary figures and images for: TFAP2C is a key regulator of intrauterine trophoblast cell invasion and deep hemochorial placentation
Source: JCI Insight. 2024 Dec 3;10(2):e186471. doi: 10.1172/jci.insight.186471 (PMC11790029; doi:10.1172/jci.insight.186471)

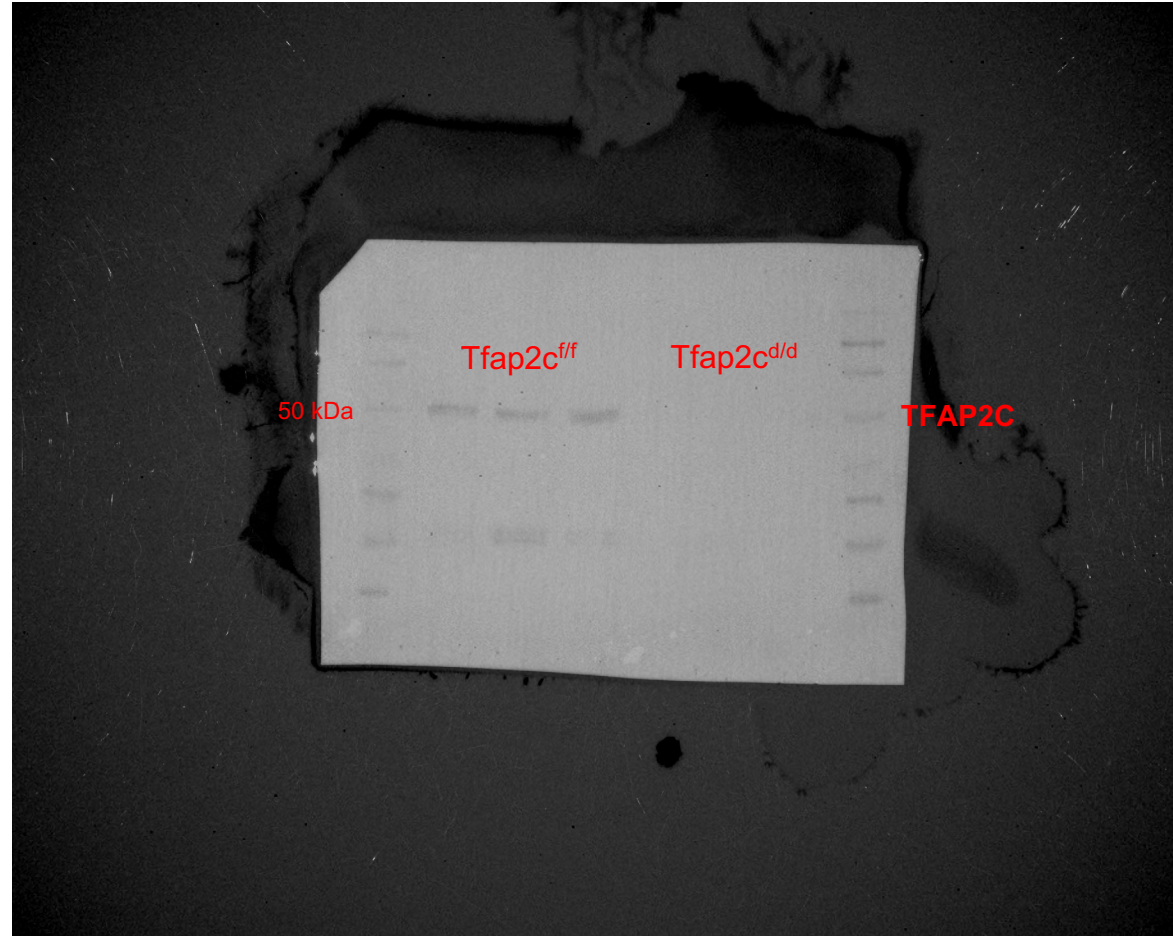

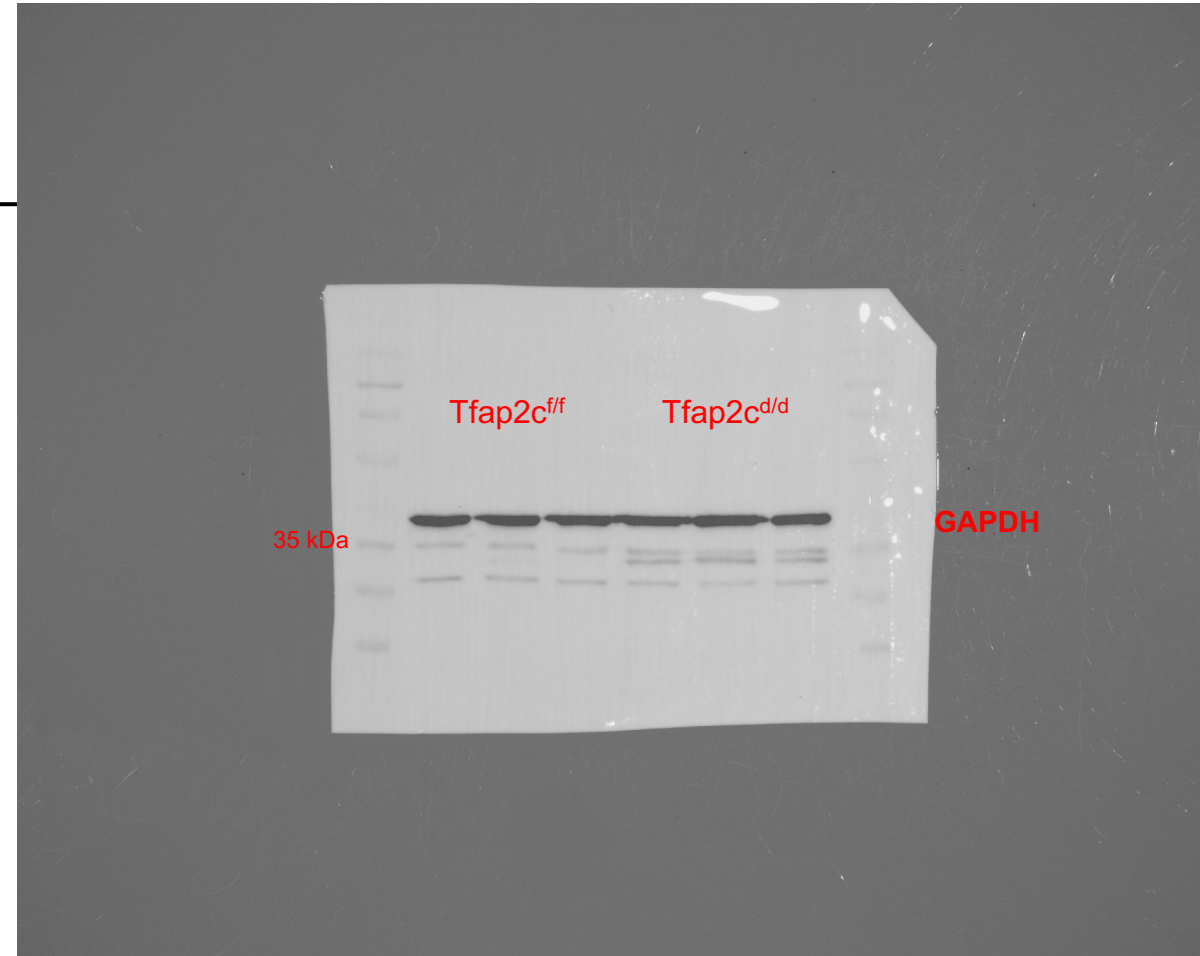

Supplement: Unedited blot and gel images [file jciinsight-10-186471-s091.pdf]
